# Sound out the impaired perfusion: Photoacoustic imaging in preclinical ischemic stroke

**DOI:** 10.3389/fnins.2022.1055552

**Published:** 2022-12-01

**Authors:** Luca Menozzi, Wei Yang, Wuwei Feng, Junjie Yao

**Affiliations:** ^1^Department of Biomedical Engineering, Duke University, Durham, NC, United States; ^2^Multidisciplinary Brain Protection Program, Department of Anesthesiology, Duke University, Durham, NC, United States; ^3^Department of Neurology, Duke University School of Medicine, Durham, NC, United States

**Keywords:** photoacoustic imaging, ischemic stroke, photoacoustic microscopy, photoacoustic computed tomography, functional brain imaging, blood oxygenation, brain perfusion

## Abstract

Acoustically detecting the optical absorption contrast, photoacoustic imaging (PAI) is a highly versatile imaging modality that can provide anatomical, functional, molecular, and metabolic information of biological tissues. PAI is highly scalable and can probe the same biological process at various length scales ranging from single cells (microscopic) to the whole organ (macroscopic). Using hemoglobin as the endogenous contrast, PAI is capable of label-free imaging of blood vessels in the brain and mapping hemodynamic functions such as blood oxygenation and blood flow. These imaging merits make PAI a great tool for studying ischemic stroke, particularly for probing into hemodynamic changes and impaired cerebral blood perfusion as a consequence of stroke. In this narrative review, we aim to summarize the scientific progresses in the past decade by using PAI to monitor cerebral blood vessel impairment and restoration after ischemic stroke, mostly in the preclinical setting. We also outline and discuss the major technological barriers and challenges that need to be overcome so that PAI can play a more significant role in preclinical stroke research, and more importantly, accelerate its translation to be a useful clinical diagnosis and management tool for human strokes.

## Introduction

Ischemic stroke occurs with a vessel blockage of blood flow in a certain region of the brain. This blood flow interruption is often a result of embolic or thrombotic occlusion of an artery within the brain. The interruption of blood flow in the brain can cause tissue damage, such as neuronal injury and death, due to a lack of vital nutrient delivery to the brain tissue. Thus, timely detection and removal of the blood clot and recanalization of the vessel are critical for acute ischemic stroke treatment (Brott and Bogousslavsky, 2000). The first-line acute stroke treatment is mechanical or pharmacologic reperfusion therapy (Green and Shuaib, 2006), and use of a clinical imaging modality such as magnetic resonance angiography (MRA) or computed tomography angiography (CTA) that can visualize the occluded vessel. These imaging modalities come at a high cost and require the use of exogenous contrast agents. Furthermore, these clinical modalities often provide only the information of blood perfusion, but lack the ability to measure blood oxygenation, the presence of specific biomolecules, or tissue viability. Thus, MRA and CTA are less used in preclinical studies of ischemic stroke. Instead, preclinical studies of ischemic stroke have heavily relied on the use of histology and behavioral testing as measures of experimental treatment efficacy. However, histology requires sacrifice of the animals and thus can only be used as an endpoint measure, and behavioral tests cannot provide concrete anatomical or physiological information. To provide a more efficient and quantitative approach to measuring stroke outcomes, various biomedical imaging technologies have recently been developed and tested for preclinical ischemic stroke studies.

**FIGURE 1:**
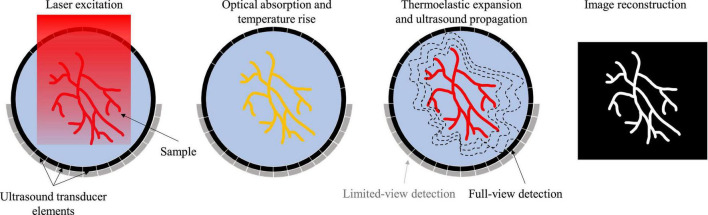
Fundamental principle of PAI.

Photoacoustic imaging (PAI) has emerged as a popular biomedical imaging modality over the past twenty years due to its intrinsic ability to combine optical contrast with acoustic detection (Xu and Wang, 2006a; Wang and Yao, 2016). The fundamental imaging principle of PAI is shown in Figure 1. A short laser pulse excites the sample, leading to the absorbers within the target to heat up *via* photothermal effect. This temperature rise results in a thermoelastic expansion and subsequently outwardly propagating ultrasound waves. In principle, any molecule absorbing light qualifies as a potential contrast in PAI, allowing for a plethora of both endogenous and exogenous contrast agents (Weber et al., 2016). Of the endogenous category, hemoglobin is the most commonly used contrast for PAI due to its abundance in biological tissues and its relatively strong optical absorption in the visible and near-infrared (NIR) light domain. With hemoglobin used as the primary contrast, PAI has been widely used as an angiographic imaging modality that produces blood vessel images in the deep tissues. While the spatial scale of PAI varies greatly from microscopic to macroscopic depending on the implementation, the contrast origin remains the same at all scales. This makes PAI a powerful research tool to investigate the same biological phenomena at vastly different scales.

Photoacoustic imaging (PAI) has two major implementations: photoacoustic microscopy (PAM) and photoacoustic computed tomography (PACT) (Yao and Wang, 2021). PAM can be further divided into optical-resolution PAM (OR-PAM) and acoustic-resolution PAM (AR-PAM). OR-PAM differentiates itself from AR-PAM based on the tight optical focusing of the excitation light. AR-PAM relies on diffuse optical excitation, while the resolution depends on the acoustic focusing of the ultrasound transducer. OR-PAM produces higher resolution images than AR-PAM but has a superficial penetration depth (∼1 mm). The macroscopic PAI implementation, PACT, utilizes diffuse light excitation and parallel acoustic detection with ultrasound transducer arrays to create tomographic images. PACT generally has a greater penetration depth (>1 cm) than PAM implementations, but lower spatial resolution (>300 μm).

Due to the high flexibility of ultrasound transducers and methods of diffuse light illumination, PACT has many different implementations. The geometry of the transducer elements, scanning pattern, central frequency and bandwidth, excitation laser wavelength and delivery method, and reconstruction technique all contribute to the resultant image quality. Depending on the design requirements of the imaging system, an optimal combination of system components can be selected. For example, while a hemispherical ultrasound transducer can produce higher spatial-resolution with minimal limited-view artifacts, it usually provides a smaller field of view than a planar or ring-shaped array with the same number of elements (Fatima et al., 2019). Furthermore, choosing a high-frequency transducer will result in high resolution images at the cost of imaging depth and increased sensitivity to the speed of sound heterogeneity. The experimental challenges of acquiring data should also be considered when designing a PACT system. For example, brain imaging with a full-ring array can be more experimentally difficult than with a hemispherical or linear-array due to the need for acoustic coupling between the head and transducer. The PACT implementation for preclinical stroke study can be optimized by considering the desired spatial and temporal resolution, field of view, detection sensitivity, cost, and animal models.

While PAI has been used in several applications in brain research (Yao and Wang, 2014a), imaging acute ischemic stroke is of particular interest, due to both the blood-sensitive nature of PAI and the severity and prevalence of ischemic stroke (Van der Worp and van Gijn, 2007). Over 795,000 people per year in the US suffer stroke, approximately 80% of which are ischemic stroke (Tsao et al., 2022). Stroke remains a leading cause of disability in the US, including language difficulty and cognitive deficits, with motor impairment being the most common complication after stroke. Many stroke survivors struggle with daily activity and have poor quality of life.

With its ability to provide both structural and functional information of the brain vessels, PAI can be a useful imaging tool in the setting of ischemic stroke. Ischemic stroke causes disrupted blood flow to a region of the brain, resulting in reduced vessel density and blood oxygenation, both of which can be readily detected by using PAI at different length scales without using any exogenous contrast agents (Li et al., 2018b). Thus, PAI of ischemic stroke has been extensively investigated and validated (Galanzha et al., 2011; Soetikno et al., 2012; Juratli et al., 2018; Das and Pramanik, 2019; Das et al., 2021). Using PAI as an alternative to MRA or CTA in evaluating post-stroke reperfusion could provide many benefits for the patients, although there are significant technical obstacles that need to be overcome.

This concise review is divided into two major sections. In the first section, we introduce the current achievements of PAI in ischemic stroke study at multiple length scales, ranging from micro vessels in small animal brains to major vessels in human brains. In the second section, we discuss the major technological challenges still faced in PAI of ischemic stroke, both in light and sound, and we present potential solutions that may translate PAI into a more powerful technology for neuroscience research.

## Applications of photoacoustic imaging in preclinical ischemic stroke research

The works presented in this review were mostly identified using the Google Scholar search engine by November 2022. The major key words used in the search engine included ischemic stroke, photoacoustic imaging, photoacoustic microscopy, photoacoustic computed tomography, and brain imaging. Over 300 papers were reviewed, wherein the relevance, novelty, and quality of the imaging systems were the main criteria when considering representative papers for inclusion.

Representative applications of PAI in ischemic stroke research in the last decade is summarized in Table 1. The included papers were chosen to represent ischemic stroke studies from single-vessel to large-animals, showing the wide range of imaging scales of PAI. Among these studies, three stroke models have been used in PAI studies: photothrombotic (PT) stroke, temporary and permanent middle cerebral artery occlusion (tMCAO and pMCAO) (Carmichael, 2005; Durukan and Tatlisumak, 2007). PT and pMCAO stroke models represent permanent ischemic stroke injury while the tMCAO model represents ischemic stroke with reperfusion.

**TABLE 1:** Summary of major publications on ischemic stroke using PAI in the past decade.

Authors	System	Resolution (lateral × axial)	Penetration	Optical wavelength	U/S detection	Stroke model	Animal model	Year
Zhu et al., 2022	OR-PAM	∼10 μm	∼1 mm	532/558 nm	40 MHz 100% bandwidth focused transducer	pMCAO	Mouse	2022
Cai et al., 2022	OR-PAM	∼24 × 45 μm	∼1 mm	720 nm	50 MHz cylindrical transducer	PT	Rat	2022
Kang et al., 2018	PACT	<1 mm	∼5 mm	700–900 nm in 10 nm intervals	128 element 5.2 MHz linear array	PT	Neonatal piglet	2022
Lv et al., 2020	PACT	∼200 μm	∼5 mm	680/750/850 nm	128 element 5 MHz hemispherical array	PT/pMCAO	Mouse	2019
Cao et al., 2018	OR-PAM	<100 μm	∼1 mm	532/558 nm	35 MHz 70% bandwidth focused transducer	tMCAO	Mouse	2018
Lin et al., 2017	OR-PAM	∼3 × 15 μm	∼1 mm	532 nm	50 MHz 100% bandwidth cylindrical transducer	PT	Mouse	2016
Deng et al., 2012	AR-PAM	∼45 × 15 μm	>1 mm	576/584/592 nm	50 MHz 70% bandwidth focused transducer	pMCAO	Rat	2012
Hu et al., 2011	OR-PAM	<100 μm	∼1 mm	563/570 nm	Cylindrical transducer	pMCAO	Mouse	2011

PT, photothrombotic; pMCAO, permanent middle cerebral artery occlusion; tMCAO, temporary middle cerebral artery occlusion.

### Imaging mini-stroke at single-vessel level

**FIGURE 2:**
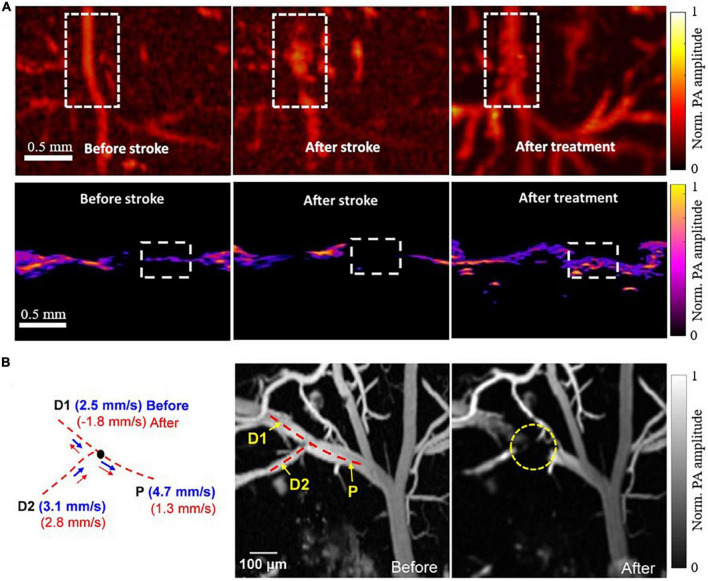
PAI of single vessel occlusions (Lin et al., 2017; Cai et al., 2022). **(A)** PAM (top row) and PACT (bottom row) images of a blood vessel before PT stroke, after PT stroke, and after experimental tPA treatment, acquired at 720nm. **(B)** PAM images of a blood vessel before and after PT stroke acquired at 532nm with corresponding blood flow velocity calculations on the left. P indicates the parent vessel of the clot, with D1 and D2 indicating two branching daughter vessels.

Taking advantage of the high spatial resolution of PAI, especially PAM, researchers have been able to investigate occlusion of a small vessel within the brain. For example, Cai et al. (2022) used both PAM and PACT to visualize single-vessel ischemic stroke using a PT stroke model in rats, as shown in Figure 2A. The PT stroke model uses a laser beam to activate a photoactive dye (e.g., Rose Bengal) and cause coagulation restricted to the illuminated region, allowing for a small beam to produce a targeted single-vessel occlusion *in vivo*. Both PA modalities were used to evaluate the performance of an experimental ischemic stroke treatment. The treatment was a photothermal-activatable liposome carrying tissue plasminogen activator (tPA), which once activated (by laser irradiation) could dissolve a blood clot and induce recanalization. The PA imaging was performed before and after stroke, and after tPA treatment, showing the healthy flow, impaired flow, and restored flow, respectively. This study displayed the feasibility of both PAM and PACT for ischemic stroke treatment monitoring, an exciting application of the imaging technology.

In another study, Lin et al. (2017) applied high-speed PAM to monitor the occurrence of mini-stroke at the capillary level in mouse models. Using a microelectromechanical system (MEMS) scanning mirror in conjunction with a high pulse repetition rate laser, they were able to image microvasculature at high spatial resolution (∼3 μm lateral and ∼15 μm axial) and high temporal resolution (400 Hz B-scan rate over 3 mm scanning range). They were able to observe the occlusion location and to measure the changes in microvascular blood flow resulting from the photothrombosis. Interestingly, their data demonstrated a vastly reduced blood flow velocity in the parent vessel, with a reversed blood flow in one of the branching daughter vessels, as shown in Figure 2B.

### Multi-scale imaging of ischemic stroke on small-animal models

**FIGURE 3:**
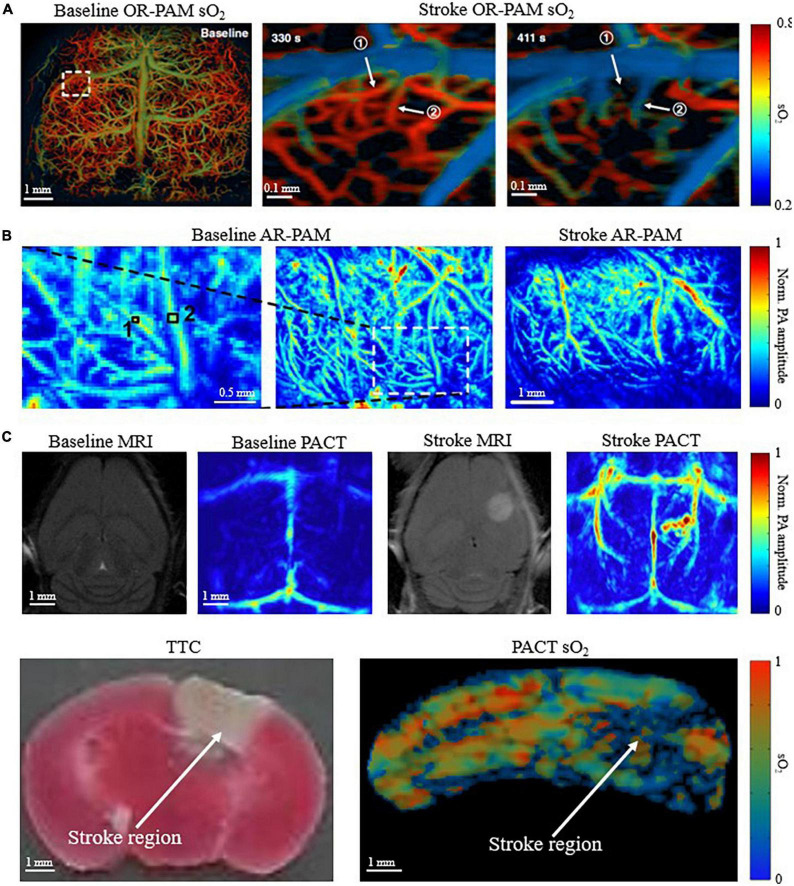
Multi-scale PAI for small-animal stroke research. **(A)** OR-PAM oxygen saturation of hemoglobin (sO_2_) image of mouse brain before stroke, 330s and 411s after stroke (skull removed) (Zhu et al., 2022). **(B)** AR-PAM image of mouse brain before and after stroke (skull removed) (Deng et al., 2012). **(C)** (Top) Baseline and post stroke MRI and corresponding PACT images of mouse brain. Infarct region in MRI corresponds with increased deoxygenated blood in PACT (scalp and skull intact) (Lv et al., 2020). (Bottom) Corresponding triphenyltetrazolium chloride (TTC) stained coronal slices and PACT sO_2_ coronal images of mouse brain. Deoxygenated region in sO_2_ image corresponds with infarct region identified by TTC (scalp and skull intact) (Menozzi et al.).

Small model animals, such as mice and rats, are commonly used in PAI technological development and biomedical applications. PAI is well suited for small-animal imaging of ischemic stroke because of the relatively thin skull, which is one of the predominant difficulties of PA and ultrasound-based brain imaging. Although not negligible, the relatively thin skull can partially mitigate the acoustic aberration and optical scattering (Liang et al., 2019; Tang and Yao, 2021). This is beneficial for achieving high imaging quality by OR-PAM (Hu et al., 2011; Yao et al., 2015), AR-PAM (Stein et al., 2009; Park et al., 2014), and PACT (Zhang et al., 2018; Lin et al., 2021) of mouse and rat brains. OR-PAM can produce the high-resolution (a few micrometers) images of ischemic stroke, such as Figure 3A. However, OR-PAM often requires removing the scalp, thinning the skull, or sometimes even installing a cranial window, which makes the modality invasive and more difficult for longitudinal studies of ischemic stroke on the same animals. OR-PAM also has superficial penetration depth (∼1 mm), since it relies primarily on the tight focusing of ballistic photons, which is undesirable for ischemic stroke studies because the injured tissue is generally not constrained to the superficial regions of the brain. On the other hand, AR-PAM utilizes diffuse photons while maintaining relatively tight acoustic focusing. This allows for improved penetration depth to ∼3-5 mm at the cost of spatial resolution (tens of micrometers) relative to OR-PAM. Figure 3B shows representative images of pre and post ischemic stroke utilizing and AR-PAM system. Of the three major implementations of PAI, PACT is the most promising for studying ischemic stroke on the whole-brain level in small-animal models, with a spatial resolution of a few hundred micrometers. Both Lv et al. (2020) and we used PACT to visualize PT stroke in mice both structurally and functionally (Figure 3C). Many small-animal PAI studies have allowed better understanding of the neuroprotective processes and hemodynamics that occur in response to ischemic stroke (Liu et al., 2015; Cao et al., 2017, 2018; Lin et al., 2017; Chen et al., 2019), and the ability to assess potential treatments for ischemic stroke (Yao et al., 2020; Cai et al., 2022).

### Deep imaging of ischemic stroke on large-animal models

**FIGURE 4:**
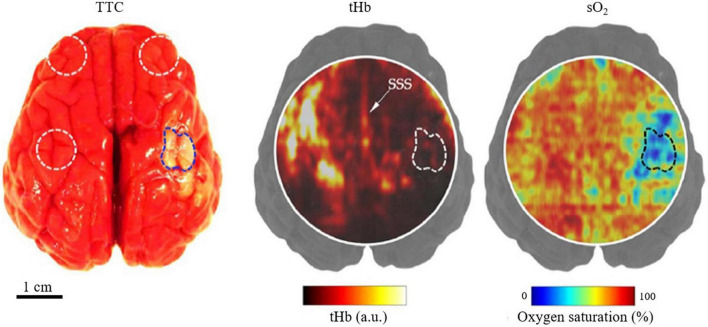
Structural and functional PACT images of a neonatal pig *in vivo* with PT stroke (Kang et al., 2022). **(Left)** TTC staining of harvested brain (post-imaging) with dotted regions indicating the regions-of-interest (ROI) for imaging. **(Middle)** PACT image of total hemoglobin concentration of a transverse brain section, showing the stroke-induced hypoperfusion, with the stroke-infarct ROI marked by the dashed line. SSS, superior sagittal sinus. **(Right)** Corresponding PA image of the blood oxygenation, showing the stroke-induced tissue hypoxia.

Photoacoustic imaging (PAI) has been successfully applied for both *ex vivo* and *in vivo* studies of ischemic stroke on large animal models. Large animal models better simulate the conditions of human brains, namely the size of the brain and thickness of the skull. Therefore, the application of PAI of ischemic stroke on large animals is a prerequisite to human stroke research. Hariri et al. tested the feasibility of transfontanelle PAI using an *ex vivo* sheep brain, with an opening in the skull mimicking the fontanelle (Hariri et al., 2017). The results showed that functional PAI through the fontanelle of a neonate brain is possible. Later, Kang et al. similarly showed the feasibility of neonatal functional PAI using an *in vivo* pig model (Kang et al., 2018). In a follow-up study, as shown in Figure 4, Kang et al. (2018) used a linear-array transducer to image neonatal pigs within 2 h following a PT stroke surgery. They found significant changes in both blood perfusion and oxygenation between injured and non-injured regions of the brain, demonstrating that PAI can be used for detecting perinatal ischemic stroke. Perinatal ischemic stroke occurs in 1 in 2300 term infants, which is 17 times more common than later in childhood or beyond (Nelson, 2007), and is a particularly compelling and clinically relevant application for PAI. The relatively small head size, the thinner bone, and the soft fontanelles of infants all help PAI achieve higher spatial resolution and larger penetration depth. These recent advances in PAI of ischemic stroke in large-animal models show a promising trend of technology advancement. Nevertheless, far fewer large-animal studies exist than small-animal studies so far, mostly due to the experimental difficulty and technology limitation. As a prerequisite to human stroke studies using PAI, more large-animal studies must be performed for extensive validation.

### Clinical translation of photoacoustic imaging for human brain imaging

**FIGURE 5:**
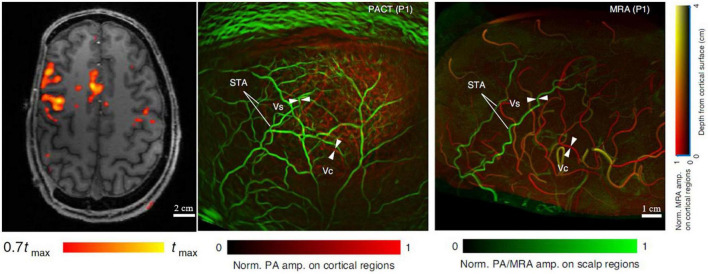
MRI, MRA, and PACT images of the brain of a hemicraniectomy patient (Na et al., 2022). **(Left)** Representative transverse MRI slice with fMRI data overlaid. **(Middle)** PACT vasculature of the brain. **(Right)** Corresponding MRA vasculature of the brain. Scalp vessel (Vs), cortical vessel (Vc), and superficial temporal arteries (STA) were used for comparison between MRA and PACT.

Ultimately PAI can be developed for use in human brain imaging, but there are several technical challenges for example, strong acoustic aberration of the thick human skull (∼6–8 mm) and the strong optical attenuation. There have been no published reports utilizing PAI to monitor ischemic stroke in humans yet. Liang et al. (2021) simulated the acoustic distortion caused by the human skull using different implementations of PACT. They found that the reflections, refractions, and mode-conversions of the human skull can introduce severe signal distortion and reconstruction artifacts in PACT, greatly reducing the resolution and quantitative accuracy of the image reconstructions. As shown in Figure 5, Na et al. imaged hemicraniectomy patients using MRA and PACT (Na et al., 2022). The results showed that the MRA and PACT can provide well-matched structural images of the human brain vasculature at depths up to 10 mm under the cortical surface. Functional MRI (fMRI) and functional PACT were also performed to monitor the brain activities in the motor and language regions in response to certain activities, such as finger tapping and passive listening. Although it has not been applied for monitoring ischemic stroke on humans yet, this recent study demonstrated that high-quality human brain imaging can be achieved with PAI technology.

## Technological challenges and solutions for photoacoustic imaging

**FIGURE 6:**
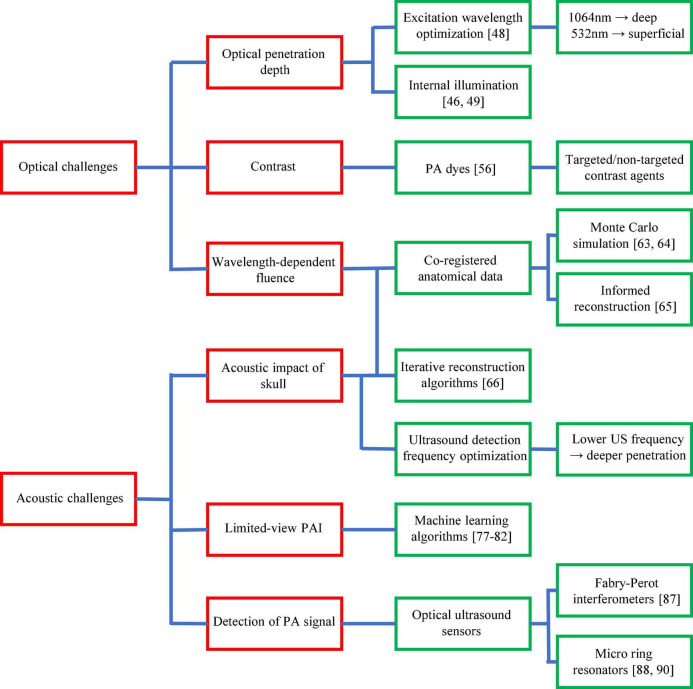
Major technological challenges of PAI in ischemic stroke research. Technological challenges are shown in red boxes with existing and potential solutions shown in green boxes.

All the above achievements in PAI of ischemic stroke have reflected the strong momentum of this exciting technology and its great potential in preclinical stroke research. However, to maximize its impact, there are still major technical challenges in PAI that need to be addressed through innovative solutions in laser engineering, data science, mathematics, and chemistry. These challenges can be broadly divided into two categories: optical challenges and acoustic challenges, as summarized in Figure 6.

### Optical challenges

#### Limited penetration depth of light due to strong optical attenuation

Skin, bone, and brain tissue are three of the most scattering biological tissues in the 500-900 nm optical wavelength range (Jacques, 2013), making light penetration deep into the brain (>1 cm depth) a difficult task. To avoid tissue damage, there exist limitations on the maximum optical energy density that can be delivered to the surface of the skin, usually guided by the laser safety standard by the American National Standards Institute (ANSI). Thus, simply increasing the excitation light energy is not a preferred option for PAI to improve its penetration depth. A few engineering solutions have been developed to improve optical penetration depth while maintaining the laser safety.

Table 2 summarizes reported illumination techniques for improving penetration depth in PAI of brains. One solution is the use of internal illumination in PAI. Laser pulses delivered within the body cavity, generally via catheter or miniaturized probe, bypass the highly absorbing layers of skin and bone, and thus can often be closer to the target being imaged. Internal illumination PAI has shown great promise for improving optical penetration depth in the animal brains through phantom experiments and *in vivo* imaging, by delivering light through the oral cavity (Lin et al., 2015; Li et al., 2018a). In a different approach, Nie et al. (2012) improved optical penetration depth by increasing the efficiency of light delivery using a photon recycler. The photon recycler was a highly reflective surface which re-delivered photons that had been scattered out of the skin surface back into the brain. Wavefront engineering is another potential solution for improving optical penetration depth while maintaining high resolution. This allows focusing light into a deeper region of a scattering medium (Kang et al., 2012; Yu et al., 2017). Optimizing the excitation wavelength can further improve optical penetration depth in PAI. For example, Xu et al. used microwave excitation to image a whole Rhesus monkey brain (Xu and Wang, 2006b). However, it should be noted that changing the excitation wavelength may change the source of contrast to other biomolecules such as water.

**TABLE 2:** Summary of illumination techniques for improving penetration depth in PAI of brains.

References	Method	Reported depth	Excitation wavelength	Target	Year
Manwar et al., 2022	Multi-angle illumination	∼2.5 cm	750 nm	*Ex vivo* sheep brain	2022
Basij et al., 2021	Dual illumination	∼3 cm	680 nm	Cervical tissue phantom	2020
Li et al., 2018a	Internal illumination	∼5 cm	1064 nm	*In vivo* mouse heart	2018
Nie et al., 2012	Photon recycler	1–2 cm	1064 nm	*Ex vivo* canine brain	2012
Xu and Wang, 2006b	Microwave excitation	∼3 cm	1.5 cm	*Ex vivo* rhesus monkey brain	2006

#### Limited optical contrast of brain functions

In the NIR excitation domain, lipids, hemoglobin, melanin, and water are the primary endogenous sources of PAI contrast (Yao and Wang, 2013). While hemoglobin can be used to probe the brain’s hemodynamics, these exogenous contrasts fall short of providing more information about the brain’s functional and molecular status, such as the neuroactivities and inflammation. Ultimately, ischemic stroke causes critical damage to the neurons in the infarct region. To precisely quantify the effect and severity of stroke, the health and function of the neurons must be evaluated, which cannot be achieved by measuring blood oxygenation alone. Many exogenous PAI contrast agents have been developed in order to resolve this issue (Pu et al., 2014; Yao and Wang, 2014b; Fu et al., 2019; Upputuri and Pramanik, 2020). Exogenous contrast agents often have stronger optical absorption and thus provide stronger PA signals and larger penetration, as well as target certain physiological events, particularly neuronal activities (Wang et al., 2016; Liu et al., 2021). In an exciting example, Shemetov et al. (2021), using an engineered NIR genetically encoded calcium indicator, simultaneously imaged hemodynamics and neuronal activity in mouse brains with PAM and fluorescence microscopy. Such targeted contrast provides a powerful new pathway for photoacoustic signal generation in the brain and can also be applied to provide more comprehensive information of the brain impaired by ischemic stroke (Song et al., 2022).

#### Inaccurate quantitative analysis due to wavelength-dependent light attenuation

Functional PAI often requires multiple excitation wavelengths to spectrally unmix the concentrations of several absorbing molecules that collectively contribute to the PA signals. Assuming a total of *n* molecules contributed to the PA signals, linear unmixing methods require at least a total of *n* images to be taken at different wavelengths, in order to solve a system of linearly independent equations with *n* unknowns. However, this commonly used linear model assumes an important oversimplification: the optical fluence at a given depth in a target strongly depends on the wavelength of light used to illuminate the target (Cox et al., 2009). This is inherently due to the wavelength-dependent optical absorption and scattering coefficients of biological tissue. Therefore, linear unmixing cannot produce accurate quantification of molecular concentrations in deep tissues. Alternatively, measurements of relative quantities can still be made and produce informative results. For example, Matsumoto et al. distinguished between arteries and veins by comparing deoxygenated blood in veins to nearby oxygenated blood in arteries (Matsumoto et al., 2018).

Although wavelength-dependent optical fluence is a pervasive problem for functional PAI, more accurate solutions do exist. Using PAI as a functionally complementary modality to MRI or computed tomography (CT) could allow for fluence-correction by simulating photon transport in tissues, using the anatomical information from MRI and CT (Wang et al., 1995; Jacques, 2014). Furthermore, iterative (or model-based) reconstruction methods, such as the algorithm proposed by Pattyn et al. (2021), can map both the optical fluence and speed of sound in the heterogeneous media (Bu et al., 2012; Huang et al., 2013; Pattyn et al., 2021), allowing for more accurate image reconstruction and spectral unmixing.

### Acoustic challenges

#### Strong acoustic impact of the skull

Reflection, refraction, and mode conversion of acoustic waves occur at boundaries of brain tissue and skull with drastically different acoustic impedance, resulting in signal loss of upward of 75% (Liang et al., 2021), which is related to the density and speed of sound of the medium (Aldrich, 2007). In ultrasound imaging, reflection is desirable during the forward propagation of the acoustic waves because it provides the image contrast. In photoacoustic imaging, however, reflection of acoustic waves is normally undesirable; the reflections reduce signal strength and introduce artifacts in the image reconstruction (Singh and Steenbergen, 2015; Nguyen et al., 2018; Liang et al., 2019). The refraction of photoacoustic waves propagating from the target to the transducer result in distortions in the reconstructed image, primarily because most reconstruction algorithms, such as the ubiquitous delay and sum (DAS) method, assume linear ultrasound transmission in a homogeneous medium (Xu and Wang, 2005). Furthermore, ultrasound propagation through the skull results in significant shear waves due to mode conversion, leading to further distortions in image reconstruction (White et al., 2006).

The most practical method for reducing the impact of the skull in PAI is to use low-frequency detection. Lower ultrasonic frequencies show less attenuation and thus greater penetration than higher frequencies, allowing for more signal to bypass the skull with less distortion. However, the use of lower frequencies also results in a reduction of spatial resolution. Co-registered anatomical information, such as from MRI or CT, could similarly improve PA reconstructions by providing segmented skull geometry (Treeby et al., 2010). This would allow the reconstruction (e.g., time reversal method) to correct for acoustic absorption, refraction, and speed-of-sound inhomogeneities between tissue layers. Skull clearing cranial windows have also shown promise in small-animal PAI to reduce both the optical and ultrasonic attenuation, improving the overall vascular brain images (Yang et al., 2016; Wang and Xi, 2021). However, these are invasive procedures, limiting their use to preclinical studies.

#### Incomplete target structure due to limited detection view

An exact reconstruction of the initial pressure distribution in PAI requires a detector that can completely surround the object being imaged, or provide a detection view of 4π. In practice, this is very challenging to implement, leading to the well-known limited-view problem in PAI that manifests as incomplete structure and reconstruction artifacts (Xu et al., 2004). The limited-view problem is augmented in brain imaging due to the size and shape of the head, adding distortions and artifacts to the image reconstruction. Deep-learning and model-based approaches have been applied to reduce the limited-view artifacts (Hauptmann et al., 2018; Guan et al., 2020; Zhang et al., 2020; Godefroy et al., 2021; Gröhl et al., 2021). Vu et al. (2020) demonstrated the use of a generative adversarial network to reduce the limited-view artifacts using simulated, phantom, and *in vivo* data. This approach allows for the reduction of the limited-view artifacts without changing the imaging system design. Even so, enlarging the detection geometry is the most effective way to mitigate the limited-view problem. For example, a 2D ring detection geometry or a hemispherical detection geometry are reported to produce superior whole-brain imaging, due to larger detection angles (Kye et al., 2022). However, using these detection arrays can introduce a higher cost of the system and also require a more complicated experimental setup. Alternatively, multiple acquisitions could be acquired at varying angles and later integrated during the image reconstruction, which, however, may result in a decreased temporal resolution and thus increased susceptibility to motion artifacts. This approach was used by Zhang et al. to image a whole mouse brain using a linear-array, in which 39 limited-view images were combined to create an enhanced image (Zhang et al., 2018). A new approach for bypassing the limited-view problem is to track sparsely distributed highly absorbing exogenous contrast as point targets as they flow through the blood vessels, though this method results in a longer scanning time as a large number of frames are needed to accumulate sufficient contrast agents in the field of view (Zhang et al., 2019).

#### Limited spatial resolution due to the band-limited ultrasound detection

Photoacoustic signals have an inherently broad bandwidth and are highly related to the target sizes, ranging from below 1 MHz to over tens of MHz (Saha and Kolios, 2011). In most PAI systems, piezo-based ultrasound transducers used in traditional ultrasound imaging are adapted as the acoustic sensor, usually due to the broad availability and convenience. However, piezo-based ultrasound transducers are not optimized to sense photoacoustic signals as the elements often have both narrow acceptance angles and narrow bandwidths. Photoacoustic imaging systems require ultrasound sensing that is more suited to the broad-band nature of a photoacoustic signal (Manwar et al., 2020).

An emerging solution is optical acoustic sensors for PAI, including amplitude-modulation-based and interferometer-based sensors. A number of studies have shown the optical acoustic sensors can usually achieve higher detection sensitives per unit sensor area, broader detection bandwidths, and larger acceptance angles, compared with traditional piezoelectric sensors (Hou et al., 2008; Li et al., 2014; Dong et al., 2016). Although optical sensors show great promise, they are mostly used as a single-element detector for either PAM or PACT (Rong et al., 2022), which has substantially limited imaging speed. Arrays of these sensors are necessary for fast PACT, but they are often difficult to fabricate with high uniformity, particularly of the resonant frequencies (Ren et al., 2021). Furthermore, resonance-based optical sensors tend to be unstable due to thermally induced resonant-frequency shifts. Improving the stability and fabrication process for optical sensors will be an enormous step forward for the field.

## Conclusion and discussion

The blood-sensitive nature of PAI makes it well suited for investigating ischemic stroke. By combining rich optical contrast with deep ultrasonic detection, PAI can be a safe alternative or complement to existing imaging modalities for studying vasculature. Furthermore, with high spatial scalability and tunable optical contrast, PAI allows for both microscopic imaging at single blood vessel level and macroscopic imaging at tissue level, providing ample metrics to monitoring ischemic stroke disease progress in timely fashion.

PAI is already a proven tool in preclinical ischemic stroke studies, particularly in small-animal stroke models. Both PAM and PACT have been used to investigate the hemodynamic processes in ischemic stroke (major publications shown in Table 1). The ability to monitor the vasculature and hemodynamics in small animals both non-invasively and longitudinally allows precise evaluation of the safety and efficacy of new treatments. As PAI technology continues to improve, more groundbreaking discoveries of the pathophysiological processes in ischemic stroke are expected in the near future.

The advancement of PAI technology in the past decade has allowed the technology to be more widely applied in imaging ischemic stroke. PAM technology has seen improvement in imaging speed, field-of-view, resolution, and sensitivity (Wang et al., 2021). These improvements have come from the application of novel high-speed multi-spectra light sources (Cho et al., 2018, 2021; Chen et al., 2022), deep learning imaging enhancement (DiSpirito et al., 2020, DiSpirito et al., 2021), innovative scanning configurations (Liu and Yao, 2018), and exogenous contrast agents (Borg and Rochford, 2018). These developments have allowed for better quantification and visualization of the hemodynamics of ischemic stroke. PACT technology has also improved in image reconstruction algorithms (Poudel et al., 2019), depth of penetration, imaging speed, and resolution (Tian et al., 2021). These advances have culminated in recent years to the use of PACT in brain imaging in large animal models and, for the first time, in humans. As PAI technology continues to develop in the coming decade, PAI-enabled advances in the understanding and treatment of ischemic stroke are expected to follow.

Although PAI has many existing clinical applications (Steinberg et al., 2019), clinical use of the technology for ischemic stroke treatment guidance and reperfusion assessment is yet to be explored. Robust solutions to the technological challenges presented in human brain imaging are a prerequisite for establishing PAI as a practical clinical tool. Promising technological advances have already been reported in attempts to solve the current problems, including the development of PA contrast agents and optical ultrasound sensors, the use of model-based image reconstructions and deep learning image enhancement, and innovative detection and illumination schemes. More rigorous evaluations of the PAI technologies on large animal stroke models are necessary before moving on to human testing.

## Author contributions

JY and LM conceived the project and wrote the manuscript. All authors reviewed and edited the manuscript.

## References

[B1] Aldrich J. E. (2007). Basic physics of ultrasound imaging. *Crit. Care Med.* 35 S131–S137. 10.1097/01.CCM.0000260624.99430.22 17446771

[B2] Basij M. Karpiouk A. Winer I. Emelianov S. Mehrmohammadi M. (2021). Dual-illumination ultrasound/photoacoustic system for cervical cancer imaging. *IEEE Photonics J.* 13:6900310. 10.1109/JPHOT.2020.3043685 33828640 PMC8023629

[B3] Borg R. E. Rochford J. (2018). Molecular photoacoustic contrast agents: Design principles & applications. *Photochem. Photobiol.* 94 1175–1209. 10.1111/php.12967 29953628 PMC6252265

[B4] Brott T. Bogousslavsky J. (2000). Treatment of acute ischemic stroke. *N. Engl. J. Med.* 343 710–722. 10.1056/NEJM200009073431007 10974136

[B5] Bu S. Liu Z. Shiina T. Kondo K. Yamakawa M. Fukutani K. (2012). Model-based reconstruction integrated with fluence compensation for photoacoustic tomography. *IEEE Trans. Biomed. Eng.* 59 1354–1363. 10.1109/TBME.2012.2187649 22345521

[B6] Cai X. Bandla A. Wang C. Liu Y-H. Chuan C. K. Xu Y. (2022). Photothermal-activatable liposome carrying tissue plasminogen activator for photoacoustic image-guided ischemic stroke treatment. *Small Struct.* 3:2100118. 10.1002/sstr.202100118

[B7] Cao R. Li J. Kharel Y. Zhang C. Morris E. Santos W. L. (2018). Photoacoustic microscopy reveals the hemodynamic basis of sphingosine 1-phosphate-induced neuroprotection against ischemic stroke. *Theranostics* 8:6111. 10.7150/thno.29435 30613286 PMC6299683

[B8] Cao R. Li J. Ning B. Sun N. Wang T. Zuo Z. (2017). Functional and oxygen-metabolic photoacoustic microscopy of the awake mouse brain. *Neuroimage* 150 77–87. 10.1016/j.neuroimage.2017.01.049 28111187 PMC5391672

[B9] Carmichael S. T. (2005). Rodent models of focal stroke: Size, mechanism, and purpose. *NeuroRx* 2. 396–409. 10.1602/neurorx.2.3.396 16389304 PMC1144484

[B10] Chen M. Jiang L. Cook C. Zeng Y. Vu T. Chen R. (2022). High-speed wide-field photoacoustic microscopy using a cylindrically focused transparent high-frequency ultrasound transducer. *Photoacoustics* 28:100417. 10.1016/j.pacs.2022.100417 36299642 PMC9589025

[B11] Chen M. Knox H. J. Tang Y. Liu W. Nie L. Chan J. (2019). Simultaneous photoacoustic imaging of intravascular and tissue oxygenation. *Opt. Lett.* 44 3773–3776. 10.1364/OL.44.003773 31368965 PMC6697144

[B12] Cho S.-W. Kang H. Park S. M. Lim G. Piao Z. Lee S. W. (2018). Optimal generation of ten individual green-to-red Raman source for wavelength-dependent real-time OR-PAM images. *IEEE J. Sel. Top. Quantum Electron.* 25 1–9. 10.1109/JSTQE.2018.2869646

[B13] Cho S.-W. Park S. M. Park B. Kim D. Y. Lee T. G. Kim B. M. (2021). High-speed photoacoustic microscopy: A review dedicated on light sources. *Photoacoustics* 24:100291. 10.1016/j.pacs.2021.100291 34485074 PMC8403586

[B14] Cox B. Laufer J. Beard P. (2009). “The challenges for quantitative photoacoustic imaging,” in *Proceedings of the photons plus ultrasound: Imaging and sensing 2009* (San Jose, CA: SPIE), 294–302. 10.1117/12.806788

[B15] Das D. Pramanik M. (2019). Combined ultrasound and photoacoustic imaging of blood clot during microbubble-assisted sonothrombolysis. *J. Biomed. Opt.* 24:121902. 10.1117/1.JBO.24.12.121902 31342692 PMC7005573

[B16] Das D. Sivasubramanian K. Rajendran P. Pramanik M. (2021). Label-free high frame rate imaging of circulating blood clots using a dual modal ultrasound and photoacoustic system. *J. Biophotonics* 14:e202000371. 10.1002/jbio.202000371 33231356

[B17] Deng Z. Wang Z. Yang X. Luo Q. Gong H. (2012). In vivo imaging of hemodynamics and oxygen metabolism in acute focal cerebral ischemic rats with laser speckle imaging and functional photoacoustic microscopy. *J. Biomed. Opt.* 17:081415. 10.1117/1.JBO.17.8.081415 23224176

[B18] DiSpirito A. III Vu T. Pramanik M. Yao J. (2021). Sounding out the hidden data: A concise review of deep learning in photoacoustic imaging. *Exp. Biol. Med.* 246 1355–1367. 10.1177/15353702211000310 33779342 PMC8243210

[B19] DiSpirito A. Li D. Vu T. Chen M. Zhang D. Luo J. (2020). Reconstructing undersampled photoacoustic microscopy images using deep learning. *IEEE Trans. Med. Imaging* 40 562–570. 10.1109/TMI.2020.3031541 33064648 PMC7858223

[B20] Dong B. Sun C. Zhang H. F. (2016). Optical detection of ultrasound in photoacoustic imaging. *IEEE Trans. Biomed. Eng.* 64 4–15. 10.1109/TBME.2016.2605451 27608445 PMC5222629

[B21] Durukan A. Tatlisumak T. (2007). Acute ischemic stroke: Overview of major experimental rodent models, pathophysiology, and therapy of focal cerebral ischemia. *Pharmacol. Biochem. Behav.* 87 179–197. 10.1016/j.pbb.2007.04.015 17521716

[B22] Fatima A. Kratkiewicz K. Manwar R. Zafar M. Zhang R. Huang B. (2019). Review of cost reduction methods in photoacoustic computed tomography. *Photoacoustics* 15:100137. 10.1016/j.pacs.2019.100137 31428558 PMC6693691

[B23] Fu Q. Zhu R. Song J. Yang H. Chen X. (2019). Photoacoustic imaging: Contrast agents and their biomedical applications. *Adv. Mater.* 31:1805875. 10.1002/adma.201805875 30556205

[B24] Galanzha E. I. Sarimollaoglu M. Nedosekin D. A. Keyrouz S. G. Mehta J. L. Zharov V. P. (2011). In vivo flow cytometry of circulating clots using negative photothermal and photoacoustic contrasts. *Cytometry A* 79 814–824. 10.1002/cyto.a.21106 21976458 PMC3366468

[B25] Godefroy G. Arnal B. Bossy E. (2021). Compensating for visibility artefacts in photoacoustic imaging with a deep learning approach providing prediction uncertainties. *Photoacoustics* 21:100218. 10.1016/j.pacs.2020.100218 33364161 PMC7750172

[B26] Green A. R. Shuaib A. (2006). Therapeutic strategies for the treatment of stroke. *Drug Discov. Today* 11 681–693. 10.1016/j.drudis.2006.06.001 16846795

[B27] Gröhl J. Schellenberg M. Dreher K. Maier-Hein L. (2021). Deep learning for biomedical photoacoustic imaging: A review. *Photoacoustics* 22:100241. 10.1016/j.pacs.2021.100241 33717977 PMC7932894

[B28] Guan S. Khan A. A. Sikdar S. Chitnis P. V. (2020). Limited-view and sparse photoacoustic tomography for neuroimaging with deep learning. *Sci. Rep.* 10:8510. 10.1038/s41598-020-65235-2 32444649 PMC7244747

[B29] Hariri A. Tavakoli E. Adabi S. Gelovani J. Avanaki M. R. (2017). “Functional photoacoustic tomography for neonatal brain imaging: Developments and challenges,” in *Proceedings of the photons plus ultrasound: Imaging and sensing 2017* (San Jose, CA: SPIE), 407–414. 10.1117/12.2254861

[B30] Hauptmann A. Lucka F. Betcke M. Huynh N. Adler J. Cox B. (2018). Model-based learning for accelerated, limited-view 3-D photoacoustic tomography. *IEEE Trans. Med. Imaging* 37 1382–1393. 10.1109/TMI.2018.2820382 29870367 PMC7613684

[B31] Hou Y. Kim J.-S. Huang S.-W. Ashkenazi S. Guo L. J. O’Donnell M. (2008). Characterization of a broadband all-optical ultrasound transducer-from optical and acoustical properties to imaging. *IEEE Trans. Ultrason. Ferroelectr. Freq. Control* 55 1867–1877. 10.1109/TUFFC.2008.870 18986929 PMC2760086

[B32] Hu S. Gonzales E. Soetikno B. Gong E. Yan P. Maslov K. (2011). “Optical-resolution photoacoustic microscopy of ischemic stroke,” in *Proceedings of the photons plus ultrasound: Imaging and sensing 2011* (Bellingham, WA: SPIE), 41–45. 10.1117/12.874366

[B33] Huang C. Wang K. Nie L. Wang L. V. Anastasio M. A. (2013). Full-wave iterative image reconstruction in photoacoustic tomography with acoustically inhomogeneous media. *IEEE Trans. Med. Imaging* 32 1097–1110. 10.1109/TMI.2013.2254496 23529196 PMC4114232

[B34] Jacques S. L. (2013). Optical properties of biological tissues: A review. *Phys. Med. Biol.* 58:R37. 10.1088/0031-9155/58/11/R37 23666068

[B35] Jacques S. L. (2014). Coupling 3D Monte Carlo light transport in optically heterogeneous tissues to photoacoustic signal generation. *Photoacoustics* 2 137–142. 10.1016/j.pacs.2014.09.001 25426426 PMC4242914

[B36] Juratli M. A. Menyaev Y. A. Sarimollaoglu M. Melerzanov A. V. Nedosekin D. A. Culp W. C. (2018). Noninvasive label-free detection of circulating white and red blood clots in deep vessels with a focused photoacoustic probe. *Biomed. Opt. Express* 9 5667–5677. 10.1364/BOE.9.005667 30460154 PMC6238938

[B37] Kang J. Boctor E. M. Adams S. Kulikowicz E. Zhang H. K. Koehler R. C. (2018). Validation of noninvasive photoacoustic measurements of sagittal sinus oxyhemoglobin saturation in hypoxic neonatal piglets. *J. Appl. Physiol.* 125 983–989. 10.1152/japplphysiol.00184.2018 29927734 PMC6335091

[B38] Kang J. Liu X. Cao S. Zeiler S. R. Graham E. M. Boctor E. M. (2022). Transcranial photoacoustic characterization of neurovascular physiology during early-stage photothrombotic stroke in neonatal piglets in vivo. *J. Neural Eng.* 18:065001. 10.1088/1741-2552/ac4596 34937013 PMC9112348

[B39] Kang M. Feng T. Wang H.-T. Li J. (2012). Wave front engineering from an array of thin aperture antennas. *Opt. Express* 20 15882–15890. 10.1364/OE.20.015882 22772278

[B40] Kye H. Song Y. Ninjbadgar T. Kim C. Kim J. (2022). Whole-body photoacoustic imaging techniques for preclinical small animal studies. *Sensors* 22:5130. 10.3390/s22145130 35890810 PMC9318812

[B41] Li H. Dong B. Zhang Z. Zhang H. F. Sun C. (2014). A transparent broadband ultrasonic detector based on an optical micro-ring resonator for photoacoustic microscopy. *Sci. Rep.* 4:4496. 10.1038/srep04496 24675547 PMC3968454

[B42] Li M. Lan B. Liu W. Xia J. Yao J. (2018a). Internal-illumination photoacoustic computed tomography. *J. Biomed. Opt.* 23:030506. 10.1117/1.JBO.23.3.030506 29573255

[B43] Li M. Tang Y. Yao J. (2018b). Photoacoustic tomography of blood oxygenation: A mini review. *Photoacoustics* 10 65–73. 10.1016/j.pacs.2018.05.001 29988848 PMC6033062

[B44] Liang B. Liu W. Zhan Q. Li M. Zhuang M. Liu Q. H. (2019). Impacts of the murine skull on high-frequency transcranial photoacoustic brain imaging. *J. Biophotonics* 12:e201800466. 10.1002/jbio.201800466 30843372 PMC11126155

[B45] Liang B. Wang S. Shen F. Liu Q. H. Gong Y. Yao J. (2021). Acoustic impact of the human skull on transcranial photoacoustic imaging. *Biomed. Opt. Express* 12 1512–1528. 10.1364/BOE.420084 33796369 PMC7984784

[B46] Lin L. Hu P. Tong X. Na S. Cao R. Yuan X. (2021). High-speed three-dimensional photoacoustic computed tomography for preclinical research and clinical translation. *Nat. Commun.* 12:882. 10.1038/s41467-021-21232-1 33563996 PMC7873071

[B47] Lin L. Xia J. Wong T. T. Li L. Wang L. V. (2015). In vivo deep brain imaging of rats using oral-cavity illuminated photoacoustic computed tomography. *J. Biomed. Opt.* 20:016019. 10.1117/1.JBO.20.1.016019 25611865 PMC4302266

[B48] Lin L. Yao J. Zhang R. Chen C. C. Huang C. H. Li Y. (2017). High-speed photoacoustic microscopy of mouse cortical microhemodynamics. *J. Biophotonics* 10 792–798. 10.1002/jbio.201600236 28009098 PMC5576888

[B49] Liu W. Yao J. (2018). Photoacoustic microscopy: Principles and biomedical applications. *Biomed. Eng. Lett.* 8 203–213. 10.1007/s13534-018-0067-2 30603203 PMC6208522

[B50] Liu X. Duan Y. Liu B. (2021). Nanoparticles as contrast agents for photoacoustic brain imaging. *Aggregate* 2 4–19. 10.1002/agt2.26

[B51] Liu Y.-H. Liao L. D. Tan S. S. H. Kwon K. Y. Ling J. M. Bandla A. (2015). Assessment of neurovascular dynamics during transient ischemic attack by the novel integration of micro-electrocorticography electrode array with functional photoacoustic microscopy. *Neurobiol. Dis.* 82 455–465. 10.1016/j.nbd.2015.06.019 26149348

[B52] Lv J. Li S. Zhang J. Duan F. Wu Z. Chen R. (2020). In vivo photoacoustic imaging dynamically monitors the structural and functional changes of ischemic stroke at a very early stage. *Theranostics* 10:816. 10.7150/thno.38554 31903152 PMC6929999

[B53] Manwar R. Kratkiewicz K. Avanaki K. (2020). Overview of ultrasound detection technologies for photoacoustic imaging. *Micromachines* 11:692. 10.3390/mi11070692 32708869 PMC7407969

[B54] Manwar R. Lara J. B. Prakash R. Ranjbaran S. M. Avanaki K. (2022). Randomized multi-angle illumination for improved linear array photoacoustic computed tomography in brain. *J. Biophotonics* 15:e202200016. 10.1002/jbio.202200016 35285133

[B55] Matsumoto Y. Asao Y. Sekiguchi H. Yoshikawa A. Ishii T. Nagae K. (2018). Visualising peripheral arterioles and venules through high-resolution and large-area photoacoustic imaging. *Sci. Rep.* 8:14930. 10.1038/s41598-018-33255-8 30297721 PMC6175891

[B56] Na S. Russin J. J. Lin L. Yuan X. Hu P. Jann K. B. (2022). Massively parallel functional photoacoustic computed tomography of the human brain. *Nat. Biomed. Eng.* 6 584–592. 10.1038/s41551-021-00735-8 34059809 PMC8630100

[B57] Nelson K. B. (2007). Perinatal ischemic stroke. *Stroke* 38 742–745. 10.1161/01.STR.0000247921.97794.5e 17261729

[B58] Nguyen H. N. Y. Hussain A. Steenbergen W. (2018). Reflection artifact identification in photoacoustic imaging using multi-wavelength excitation. *Biomed. Opt. Express* 9 4613–4630. 10.1364/BOE.9.004613 30319890 PMC6179390

[B59] Nie L. Cai X. Maslov K. I. Garcia-Uribe A. Anastasio M. A. Wang L. V. (2012). Photoacoustic tomography through a whole adult human skull with a photon recycler. *J. Biomed. Opt.* 17:110506. 10.1117/1.JBO.17.11.110506 23123972 PMC3487537

[B60] Park S. Lee C. Kim J. Kim C. (2014). Acoustic resolution photoacoustic microscopy. *Biomed. Eng. Lett.* 4 213–222. 10.1007/s13534-014-0153-z

[B61] Pattyn A. Mumm Z. Alijabbari N. Duric N. Anastasio M. A. Mehrmohammadi M. (2021). Model-based optical and acoustical compensation for photoacoustic tomography of heterogeneous mediums. *Photoacoustics* 23:100275. 10.1016/j.pacs.2021.100275 34094852 PMC8167150

[B62] Poudel J. Lou Y. Anastasio M. A. (2019). A survey of computational frameworks for solving the acoustic inverse problem in three-dimensional photoacoustic computed tomography. *Phys. Med. Biol.* 64:14TR01. 10.1088/1361-6560/ab2017 31067527

[B63] Pu K. Shuhendler A. J. Jokerst J. V. Mei J. Gambhir S. S. Bao Z. (2014). Semiconducting polymer nanoparticles as photoacoustic molecular imaging probes in living mice. *Nat. Nanotechnol.* 9 233–239. 10.1038/nnano.2013.302 24463363 PMC3947658

[B64] Ren D. Sun Y. Shi J. Chen R. (2021). A review of transparent sensors for photoacoustic imaging applications. *Photonics* 8:324. 10.3390/photonics8080324 31434241

[B65] Rong Q. Lee Y. Tang Y. Vu T. Taboada C. Zheng W. (2022). High-frequency 3D photoacoustic computed tomography using an optical microring resonator. *BME Front.* 2022:9891510. 10.34133/2022/9891510 PMC9933894 36818003

[B66] Saha R. K. Kolios M. C. (2011). A simulation study on photoacoustic signals from red blood cells. *J. Acoust. Soc. Am.* 129 2935–2943. 10.1121/1.3570946 21568396

[B67] Shemetov A. A. Monakhov M. V. Zhang Q. Canton-Josh J. E. Kumar M. Chen M. (2021). A near-infrared genetically encoded calcium indicator for in vivo imaging. *Nat. Biotechnol.* 39 368–377. 10.1038/s41587-020-0710-1 33106681 PMC7956128

[B68] Singh M. K. A. Steenbergen W. (2015). Photoacoustic-guided focused ultrasound (PAFUSion) for identifying reflection artifacts in photoacoustic imaging. *Photoacoustics* 3 123–131. 10.1016/j.pacs.2015.09.001 31467843 PMC6713059

[B69] Soetikno B. Hu S. Gonzales E. Zhong Q. Lee J. (2012). “Vessel segmentation analysis of ischemic stroke images acquired with photoacoustic microscopy,” in *Proceedings of the photons plus ultrasound: Imaging and sensing 2012* (Bellingham, WA: SPIE), 765–769. 10.1117/12.911089

[B70] Song G. Ni D. Bu W. Zhou Q. Fan W. Wu Y. (2022). MnCO3@ BSA-ICG nanoparticles as a magnetic resonance/photoacoustic dual-modal contrast agent for functional imaging of acute ischemic stroke. *Biochem. Biophys. Res. Commun.* 614 125–131. 10.1016/j.bbrc.2022.04.143 35580541

[B71] Stein E. W. Maslov K. Wang L. V. (2009). Noninvasive, in vivo imaging of the mouse brain using photoacoustic microscopy. *J. Appl. Phys.* 105:102027. 10.1063/1.3116134 PMC2719465 19657402

[B72] Steinberg I. Huland D. M. Vermesh O. Frostig H. E. Tummers W. S. Gambhir S. S. (2019). Photoacoustic clinical imaging. *Photoacoustics* 14 77–98. 10.1016/j.pacs.2019.05.001 31293884 PMC6595011

[B73] Tang Y. Yao J. (2021). 3D Monte Carlo simulation of light distribution in mouse brain in quantitative photoacoustic computed tomography. *Quant. Imaging Med. Surg.* 11:1046. 10.21037/qims-20-815 33654676 PMC7829164

[B74] Tian C. Zhang C. Zhang H. Xie D. Jin Y. (2021). Spatial resolution in photoacoustic computed tomography. *Rep. Prog. Phys.* 84:036701. 10.1088/1361-6633/abdab9 33434890

[B75] Treeby B. E. Zhang E. Z. Cox B. T. (2010). Photoacoustic tomography in absorbing acoustic media using time reversal. *Inverse Probl.* 26:115003. 10.1088/0266-5611/26/11/115003

[B76] Tsao C. W. Aday A. W. Almarzooq Z. I. Alonso A. Beaton A. Z. Bittencourt M. S. (2022). Heart disease and stroke statistics–2022 update: A report from the American heart association. *Circulation* 145 e153–e639. 10.1161/CIR.0000000000001052 35078371

[B77] Upputuri P. K. Pramanik M. (2020). Recent advances in photoacoustic contrast agents for in vivo imaging. *Wiley Interdiscip. Rev. Nanomed. Nanobiotechnol.* 12:e1618. 10.1002/wnan.1618 32027784

[B78] Van der Worp H. B. van Gijn J. (2007). Acute ischemic stroke. *N. Engl. J. Med.* 357 572–579. 10.1056/NEJMcp072057 17687132

[B79] Vu T. Li M. Humayun H. Zhou Y. Yao J. (2020). A generative adversarial network for artifact removal in photoacoustic computed tomography with a linear-array transducer. *Exp. Biol. Med.* 245 597–605. 10.1177/1535370220914285 32208974 PMC7153213

[B80] Wang D. Wu Y. Xia J. (2016). Review on photoacoustic imaging of the brain using nanoprobes. *Neurophotonics* 3:010901. 10.1117/1.NPh.3.1.010901 PMC4699324 26740961

[B81] Wang K. Li C. Chen R. Shi J. (2021). Recent advances in high-speed photoacoustic microscopy. *Photoacoustics* 24:100294. 10.1016/j.pacs.2021.100294 34458095 PMC8379700

[B82] Wang L. V. Yao J. (2016). A practical guide to photoacoustic tomography in the life sciences. *Nat. Methods* 13 627–638. 10.1038/nmeth.3925 27467726 PMC4980387

[B83] Wang L. Jacques S. L. Zheng L. (1995). MCML–monte carlo modeling of light transport in multi-layered tissues. *Computer Methods Programs Biomed.* 47 131–146. 10.1016/0169-2607(95)01640-F 7587160

[B84] Wang Y. Xi L. (2021). Chronic cranial window for photoacoustic imaging: A mini review. *Vis. Comput. Ind. Biomed. Art* 4 1–9. 10.1186/s42492-021-00081-1 34037873 PMC8155166

[B85] Weber J. Beard P. C. Bohndiek S. E. (2016). Contrast agents for molecular photoacoustic imaging. *Nat. Methods* 13 639–650. 10.1038/nmeth.3929 27467727

[B86] White P. J. Clement G. T. Hynynen K. (2006). Longitudinal and shear mode ultrasound propagation in human skull bone. *Ultrasound Med. Biol.* 32 1085–1096. 10.1016/j.ultrasmedbio.2006.03.015 16829322 PMC1560344

[B87] Xu M. Wang L. V. (2005). Universal back-projection algorithm for photoacoustic computed tomography. *Phys. Rev. E Stat. Nonlin. Soft Matter Phys.* 71(1 Pt 2):016706. 10.1103/PhysRevE.71.016706 15697763

[B88] Xu M. Wang L. V. (2006a). Photoacoustic imaging in biomedicine. *Rev. Sci. Instrum.* 77:041101. 10.1063/1.2195024

[B89] Xu Y. Wang L. V. (2006b). Rhesus monkey brain imaging through intact skull with thermoacoustic tomography. *IEEE Trans. Ultrason. Ferroelectr. Freq. Control* 53 542–548. 10.1109/TUFFC.2006.1610562 16555762

[B90] Xu Y. Wang L. V. Ambartsoumian G. Kuchment P. (2004). Reconstructions in limited-view thermoacoustic tomography. *Med. Phys.* 31 724–733. 10.1118/1.1644531 15124989

[B91] Yang X. Zhang Y. Zhao K. Zhao Y. Liu Y. Gong H. (2016). Skull optical clearing solution for enhancing ultrasonic and photoacoustic imaging. *IEEE Trans. Med. Imaging* 35 1903–1906. 10.1109/TMI.2016.2528284 26886977

[B92] Yao J. Wang L. V. (2013). Photoacoustic microscopy. *Laser Photon. Rev.* 7 758–778. 10.1002/lpor.201200060 24416085 PMC3887369

[B93] Yao J. Wang L. V. (2014a). Photoacoustic brain imaging: From microscopic to macroscopic scales. *Neurophotonics* 1:011003. 10.1117/1.NPh.1.1.011003 25401121 PMC4232215

[B94] Yao J. Wang L. V. (2014b). Sensitivity of photoacoustic microscopy. *Photoacoustics* 2 87–101. 10.1016/j.pacs.2014.04.002 25302158 PMC4182819

[B95] Yao J. Wang L. V. (2021). Perspective on fast-evolving photoacoustic tomography. *J. Biomed. Opt.* 26:060602. 10.1117/1.JBO.26.6.060602 34196136 PMC8244998

[B96] Yao J. Wang L. Yang J. M. Maslov K. I. Wong T. T. Li L. (2015). High-speed label-free functional photoacoustic microscopy of mouse brain in action. *Nat. Methods* 12 407–410. 10.1038/nmeth.3336 25822799 PMC4428901

[B97] Yao M. Shi X. Zuo C. Ma M. Zhang L. Zhang H. (2020). Engineering of SPECT/photoacoustic imaging/antioxidative stress triple-function nanoprobe for advanced mesenchymal stem cell therapy of cerebral ischemia. *ACS Appl. Mater. Interfaces* 12 37885–37895. 10.1021/acsami.0c10500 32806884

[B98] Yu Z. Li H. Lai P. (2017). Wavefront shaping and its application to enhance photoacoustic imaging. *Appl. Sci.* 7:1320. 10.3390/app7121320

[B99] Zhang H. Li H. Nyayapathi N. Wang D. Le A. Ying L. (2020). A new deep learning network for mitigating limited-view and under-sampling artifacts in ring-shaped photoacoustic tomography. *Comput. Med. Imaging Graph.* 84:101720. 10.1016/j.compmedimag.2020.101720 32679469

[B100] Zhang P. Li L. Lin L. Hu P. Shi J. He Y. (2018). High-resolution deep functional imaging of the whole mouse brain by photoacoustic computed tomography in vivo. *J. Biophotonics* 11:e201700024. 10.1002/jbio.201700024 28635056 PMC5777675

[B101] Zhang P. Li L. Lin L. Shi J. Wang L. V. (2019). In vivo superresolution photoacoustic computed tomography by localization of single dyed droplets. *Light Sci. Appl.* 8 1–9. 10.1038/s41377-019-0147-9 30962922 PMC6445830

[B102] Zhu X. Huang Q. DiSpirito A. Vu T. Rong Q. Peng X. (2022). Real-time whole-brain imaging of hemodynamics and oxygenation at micro-vessel resolution with ultrafast wide-field photoacoustic microscopy. *Light Sci. Appl.* 11:138. 10.1038/s41377-022-00836-2 35577780 PMC9110749

